# CD68- and CD163-positive tumor-associated macrophages in triple negative cancer of the breast

**DOI:** 10.1007/s00428-020-02855-z

**Published:** 2020-06-30

**Authors:** Tsengelmaa Jamiyan, Hajime Kuroda, Rin Yamaguchi, Akihito Abe, Mitsuhiro Hayashi

**Affiliations:** 1 Department of Diagnostic Pathology, Dokkyo Medical University, 880 Kitakobayashi, Mibu, Shimotsuga District, Tochigi, 321-0293 Japan; 2 Department of Pathology and Forensic medicine, Mongolian National University of Medical Sciences, Jamyan St 3, Ulaanbaatar, 14210 Mongolia; 3 Department of Diagnostic Pathology, Tokyo Women’s Medical University, Medical Center East, 2-1-10 Nishiogu, Arakawa-ku, Tokyo 116-8567, Japan; 4 Department of Pathology and Laboratory Medicine, Kurume University Medical Center, 155-1 Kokubumachi, Kurume, Fukuoka, 839-0863 Japan; 5 Breast center, Dokkyo Medical University, 880 Kitakobayashi, Mibu, Shimotsuga District, Tochigi 321-0293, Japan; 6 Department of Surgery II, Dokkyo Medical University, 880 Kitakobayashi, Mibu, Shimotsuga District, Tochigi, 321-0293 Japan

**Keywords:** Breast, Triple-negative cancer, Tumor-associated macrophages, CD68, CD163

## Abstract

Tumor-associated macrophages (TAMs) have recently been reported as an important factor in tumor growth and the progression of cancer. The prognostic significance of localizations and densities of TAMs in triple negative cancer (TNC) of the breast is not well understood. The aim of this study was to assess the localizations and densities of the TAMs subtype in TNC and examine their clinicopathological features. The study was based on 107 TNC cases operated on at Dokkyo Medical University Hospital using the pan-macrophage marker CD68 and the M2 macrophage marker CD163 in the tumor stroma (TS) and tumor nest (TN), respectively, and examined the clinicopathological significance. Multivariate Cox regression analyses revealed that age and CD163+ TAMs in both the TS and TN were independent prognostic factors for relapse-free survival and overall survival. No correlation was found between the number of CD68+ cells or the CD163/CD68 ratio either in TS or TN, or clinicopathological features. Our study found that infiltration of CD163+ TAMs, rather than CD68+, in both TS and TN was associated with poor prognosis in TNC patients by multivariate analysis. This suggests that CD163+ TAMs may affect the prognosis of TNC by not only regulating the immune reaction by TAMs in TS, but also because of their direct influence on TN.

## Introduction

Tumor-associated macrophages (TAMs) have recently been reported as an important factor in tumor growth and the progression of cancer. Recently, two processes were proposed for TAMs activation: Classically-activated type 1 (M1-like) macrophages and alternatively-activated type 2 (M2-like) macrophages. M1-like macrophages, characterized by CD68 expression, produce free radicals that can lead to DNA damage with the potential to contribute to tumoricidal activity [1]. In contrast, M2-like macrophages, characterized by both CD68 and CD163 expression, are considered to promote tumor growth and metastasis by releasing chemokines, which are inflammatory growth factors [2, 3]. Previous studies confirmed that TAMs are associated with cancer survival in several organs such as hepatoma [4], gastric cancer [5], and lung cancer [6]. In breast cancer, several studies have demonstrated that TAMs are related to hormonal status, stage, lymph node (LN) status, and prognosis [7–10]. Therefore, TAMs in different regions and at different densities may have different prognostic value in breast cancer. In general, triple-negative cancer (TNC) is characterized by a lack of expression of the estrogen receptor (ER), progesterone receptor (PgR) and human epidermal growth factor receptor 2 (HER2) protein; this type is well known to have a poor prognosis [11, 12]. However, we should note that TNCs do not always correlate with poor prognosis. Therefore, to confirm the association of TAMs and TNC, a larger cohort using different statistical methods should be evaluated. Moreover, the prognostic significance of localizations and densities of CD68+ and CD163+ TAMs in TNC is not well understood. The aim of this study was to assess the localizations and densities of the macrophage markers CD68+ and CD163+ TAMs in TNC and examine their clinicopathological features.

## Materials and methods

### Patients

The study was based on 107 TNC cases operated on at Dokkyo Medical University Hospital between 2006 and 2018. Patient and tumor characteristics, including patient age at the time of diagnosis, tumor size, histologic grade, LN status, and follow-up data, were determined from patients’ medical records and pathology reports. Relapse-free survival (RFS) was defined as the number of months from surgical resection to the development of documented relapse, including recurrence or distant metastasis. Overall survival (OS) was recorded from the date of curative surgery to the date of breast cancer-specific death.

The present study was approved by the Ethics Committee of Dokkyo Medical University (Tochigi, Japan; registration number: 28009) and was conducted according to the Declaration of Helsinki.

### Immunohistochemistry (IHC)

Surgical sections were immunostained for ER (clone SP1, Novocastra (Leica), prediluted, nuclear), PgR (clone 1E2, Novocastra (Leica), prediluted, nuclear), HER2 (clone 4B5, Roche (VENTANA), prediluted, membranous), CD68 (CD68, clone PG-M1, Dako (Agilent), 1:50), and CD163 (CD163, clone 10D6, Novocastra (Leica), 1:50). Counterstaining was performed with hematoxylin. The percentages of nuclei stained for ER and PgR were calculated, as stated by the guideline, and a patient was considered to be “positive” if the breast tumor contained at least 1% positive cells [13]. HER2 status was assessed according to the guidelines defined by the American Society of Clinical Oncology/College of American Pathologists [14]. We estimated the TILs on hematoxylin and eosin (H&E) stained sections according to the criteria proposed by the International Immuno-Oncology Biomarkers Working Group [15]. TILs levels were categorized as high (≥ 30%) and low (< 30%) adopting previously validated cut-offs [16].

TAMs were evaluated by adapting the previously reported hotspot quantitative method [7, 10, 17–19]. The CD68+ and CD163+ staining was assessed by counting the number of positive macrophages. TAMs were scored as the infiltration density of CD68+ or CD163+ cells with a macrophage morphology that showed strong membranous or cytoplasmic staining. Each specimen was screened at low magnification (× 100), and the five areas with the greatest number of positively stained cells (hot spot area) were selected for further analysis. The mean macrophage count in these areas for each case was estimated at high power (× 400) magnification. The CD68+ and CD163+ macrophages were counted in the tumor stroma (TS) and tumor nest (TN) separately. The definition of TS in this study was the stromal tissue surrounding the tumor nest. TAMs in TN were defined as intraepithelial tumor-infiltrating macrophages. For statistical analyses, the number of positive cells was divided into lower and higher groups based on cut-off points according to the median. As a result, the cut-off for CD68 in TS was 26.2, CD68 in TN was 11.2, CD163 in TS was 26.6, CD163 in TN was 8.6 CD163/CD68 in TS was 1.0, CD163/CD68 in TN was 0.99 (Table 1). Two pathologists (TJ and HK) did the evaluations without access to any clinical information.

**Table 1:** Distribution pattern of TAMs in TNC

Variables	Mean	SE	Median	Range
CD68+ TAMs				
Tumor stroma	28.85	1.2	26.2	5–96.2
Tumor nest	13.36	0.74	11.2	1.6–43
CD163+ TAMs				
Tumor stroma	29.58	1.45	26.6	4.2–78.2
Tumor nest	10.58	0.88	8.6	0–44.8
Ratio of CD163 and CD68				
Tumor stroma	0.99	0.007	1	0.79–1.2
Tumor nest	0.98	0.005	0.99	0.84–1.12

*TAMs* tumor-associated macrophages*, TNC* triple-negative cancer, *SE* standard error

### Statistical analysis

Spearman’s Rho and *χ2* tests were used to compare CD68 and CD163 expression and patient and tumor characteristics. Kaplan-Meier analysis and log-rank tests were used to illustrate differences in RFS and OS according to CD163 and CD68 expression. Cox regression proportional hazards models were used to estimate hazard ratios (HR) for death from breast cancer according to CD68 and CD163 expression in both uni- and multivariate analysis. Covariates with a *P* value ≤ 0.05 in the univariate analysis were included in the multivariate analysis. All statistical tests were two-sided and *P* ≤ 0.05 was considered significant. Statistical analysis was performed using IBM SPSS Statistics 25 (IBM, Armonk, NY, USA).

## Results

**Fig. 1:**
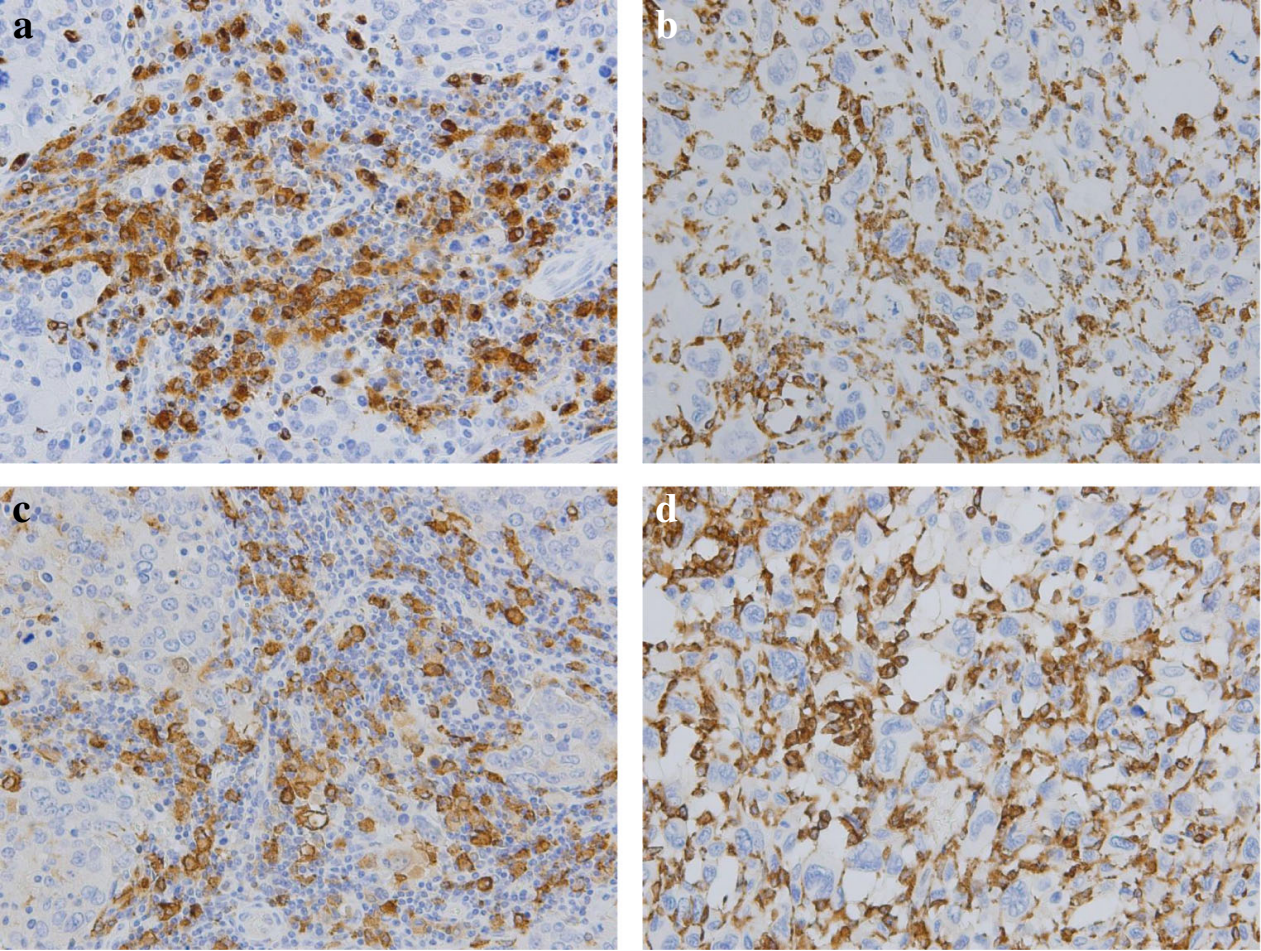
Immunohistochemical staining for the infiltration of CD68+ tumor-associated macrophages (TAMs) and CD163+ TAMs in triple-negative cancer (TNC) of the breast. Representative images of high density CD68+ staining (**a**, **b**) and CD163+ staining (**c**, **d**) in tumor stroma and tumor nest. (original magnification, ×200)

The CD68 and CD163 expressions in TS and TN were determined for all 107 samples. CD68+ (Fig. 1a, b) and CD163+ (Fig. 1c, d) macrophages were detected in both the TS and TN of TNC. The relationship between the density of TAMs (CD68+, or CD163+) and clinicopathological features is shown in Table 2. The study demonstrated that a high density of CD68+ TAMs in both TS and TN was significantly associated with larger tumor size (*p* = 0.036; *p* = 0.004). Whereas a high density of CD163+ TAMs in TN was also significantly related to larger tumor size (*p* = 0.002), however, not in TS (*p* = 0.634). Moreover, a high density of CD163+ TAMs in both TS and TN were correlated with higher histological grade (*p* < 0.001; *p* = 0.010), higher recurrence rate (*p* < 0.001; *p =* 0.004), and higher breast cancer mortality (*p* = 0.004, *p* = 0.012). In contrast, no significant correlations were found between the infiltration densities of TAMs (CD68+, CD163+, CD163/CD68 ratio) and TILs in both TS (*p* = 0.635, *p* = 0.382, and *p* = 0.382, respectively) or TN (*p* = 0.635, *p* = 0.861, and *p* = 0.670, respectively). No correlation was found between the CD163/CD68 ratios for either TS or TN or in terms of clinicopathological features.

**Table 2:** Clinicopathological features of triple-negative cancer (TNC) and the status of TAMs

Clinicopathological features	CD68						CD163						CD163/CD68					
	Tumor stroma			Tumor nest			Tumor stroma			Tumor nest			Tumor stroma			Tumor nest		
	Low	High	*p* value	Low	High	*p* value	Low	High	*p* value	Low	High	*p* value	Low	High	*p* value	Low	High	*p* value
Age (years)			*0.364*			*0.719*			*0.183*			*0.541*			*0.627*			*0.072*
< 50	14	18		17	15		13	19		15	17		17	15		11	21	
≥ 50	40	35		37	38		41	34		40	35		36	39		40	35	
Tumor size (cm)			0.036*			0.004*			*0.634*			0.002*			*0.634*			*0.208*
≤ 2	40	29		42	27		36	33		43	26		33	36		36	33	
> 2	14	24		12	26		18	20		12	26		20	18		15	23	
Histological grade			*0.063*			*0.564*			< 0.001*			0.010*			*0.26*			*0.924*
I and II	20	11		17	14		25	6		22	9		18	13		15	16	
III	34	42		37	37		29	47		33	43		35	41		36	40	
Histological type			*0.501*			*0.629*			*0.298*			*0.148*			*0.298*			*0.408*
IBC-NST	39	38		37	40		39	38		38	39		39	38		38	39	
IBC-NST with medullary pattern	3	8		5	6		4	7		4	7		7	4		3	8	
ca with apocrine differentiation	6	4		7	3		7	3		9	1		3	7		5	5	
Metaplastic carcinoma	4	2		4	2		2	4		3	3		4	2		3	3	
ILC	1	1		1	1		2	0		1	1		0	2		2	0	
IMP	1	0		0	1		0	1		0	1		0	1		0	1	
Lymph node status			*0.794*			*0.789*			*0.115*			*0.054*			*0.212*			*0.499*
Absent	31	34		33	32		38	27		39	26		37	28		29	36	
Present	15	15		14	16		11	19		10	20		13	17		17	13	
N/A	7	5		7	5		5	7		6	6		4	8				
TILs			*0.635*			*0.635*			*0.382*			*0.861*			*0.382*			*0.670*
Low (≤ 30%)	35	32		35	32		36	31		34	33		31	36		33	34	
High (> 30%)	19	21		19	21		18	22		21	19		22	18		18	22	
Recurrence			*0.053*			*0.185*			< 0.001*			0.004*			*0.185*			*0.485*
No	45	35		43	37		46	34		46	34		43	37		35	45	
Yes	6	13		7	12		1	18		4	15		7	12		10	9	
Breast cancer mortality			*0.179*			*0.970*			0.004*			0.012*			*0.059*			*0.736*
No	47	40		44	43		46	41		48	39		47	40		39	48	
Yes	4	8		6	6		1	11		2	10		3	9		6	6	

*TNC* triple-negative cancer, *TAMs* tumor-associated macrophages*, IBC-NST* invasive breast carcinoma of no special type, *ca* carcinoma, *ILC* invasive lobular carcinoma, *IMP* invasive micropapillary carcinoma, *N/A* not applicable, *TILs* tumor-infiltrating lymphocytes

*p value is significant

*χ2 test

Univariate and multivariate Cox regression analysis of RFS and OS were performed using clinicopathological prognostic factors and expressions of CD68 and CD163 (Table 3). Multivariate Cox regression analyses revealed that age and CD163+ TAMs in both TS and TN were independent prognostic factors for RFS (HR = 0.164, 95% CI 0.048–0.560, *p* = 0.004; HR = 9.059, 95% CI 1.160–70.76, *p* = 0.036; HR = 4.476, 95% CI 1.028–22.08, *p* = 0.046) and OS (HR = 0.095, 95% CI 0.024–0.374, *p* = 0.001; HR = 10.69, 95% CI 1.313–87.18, *p* = 0.027; HR = 5.017, 95% CI 1.065–23.64, *p* = 0.041).

**Table 3:** Univariate and multivariate Cox regression analyses for relapse-free survival (RFS) and overall survival (OS) of triple-negative cancer (TNC)

Clinicopathological features	Univariate analysis		Multivariate analysis		Univariate analysis		Multivariate analysis	
	RFS HR (95% CI)	*p* value	RFS HR (95% CI)	*p* value	OS HR (95% CI)	*p* value	OS HR (95% CI)	*p* value
Age (< 50 vs. ≥ 50)	0.169 (0.051–0.568)	0.004*	0.164 (0.048–0.560)	0.004*	0.128 (0.037–0.437)	0.001*	0.095 (0.024–0.374)	0.001*
Tumor size (2 cm vs. > 2 cm)	2.535 (0.809–7.936)	*0.110*			3.012 (0.962–9.431)	*0.058*		
Histological grade (I, II vs. III)	2.233 (0.488–10.22)	*0.300*			2.274 (0.497–10.411)	*0.29*		
Histological type (IBC-NST vs. other types)	0.770(0.413–1.438)	*0.413*			0.799(0.431–1.481)	*0.476*		
Lymph node status (absent vs. present)	1.683 (0.818–3.465)	*0.158*			1.820 (0.868–3.816)	*0.113*		
TILs (low vs. high)	0.449(0.121–1.660)	*0.230*			0.433(0.117–1.606)	*0.211*		
TS CD68 (low vs. high)	1.021 (0.984–1.059)	*0.268*			2.619 (0.783–8.756)	*0.118*		
TN CD68 (low vs. high)	0.969 (0.312–3.008)	*0.957*			0.938 (0.302–2.911)	*0.912*		
TS CD163 (low vs. high)	11.50 (1.481–89.29)	0.020*	9.059 (1.160–70.76)	0.036*	10.597(1.366–82.192)	0.024*	10.69 (1.313–87.18)	0.027*
TN CD163 (low vs. high)	4.952 (1.084–22.61)	0.039*	4.476 (1.028–22.08)	0.046*	4.735 (1.037–21.623)	0.045*	5.017 (1.065–23.64)	0.041*
TS CD163/CD68 (low vs. high)	2.972 (0.804–10.98)	*0.102*			2.829 (0.765–10.45)	*0.119*		
TN CD163/CD68 (low vs. high)	0.853 (0.275–2.646)	*0.783*			0.924 (0.298–2.868)	*0.892*		

Multivariate Cox regression analyses were performed for all potential variables that were significantly associated with survival in univariate analysis. *RFS* relapse-free survival and *OS* overall survival. *TNC* triple-negative cancer, *HR* hazard ratio, *CI* confidence interval, *IBC-NST* invasive breast carcinoma of no special type, *TILs* tumor-infiltrating lymphocytes, *TS* tumor stroma, *TN* tumor nest

*p value is significant

**Fig. 2:**
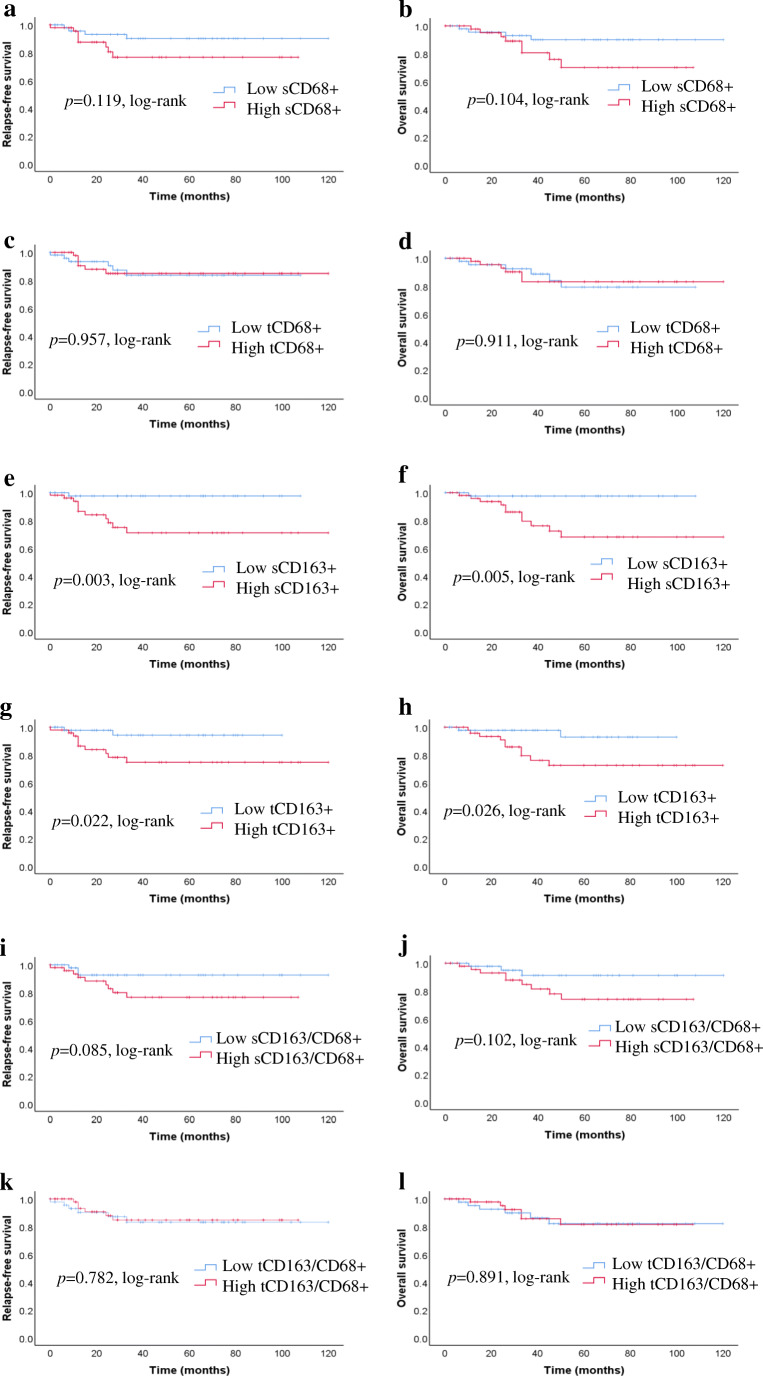
Prognostic significance of TAMs in breast cancer. Kaplan–Meier curves for relapse-free survival (RFS) and overall survival (OS) were stratified by the median values as the cut-off for prognostic evaluation and divided into low or high TAMs variable subsets. CD68+ TAMs did not demonstrate prognostic significance for RFS (**a**, **c**) or OS (**b**, **d**) in tumor stroma (TS) and tumor nest (TN). High density of CD163+ TAMs in TS and TN were associated with poor RFS (**e**, **g**) and OS (**f**, **h**). The RFS (**i**, **k**) and OS (**j**, **l**) curves according to the infiltration density of CD163/CD68+ ratios in TS and TN

We investigated survival rate with regard to the different expressions of TAMs status using the Kaplan-Meier method and log-rank test. No correlation was found in the higher CD68+ TAMs density in both TS and TN with RFS (*p* = 0.119; *p* = 0.957) or OS (*p* = 0.104; *p* = 0.911) (Fig. 2a, b, c, and d). A higher CD163+ TAMs density in both TS and TN was correlated with unfavorable RFS (*p* = 0.003; *p* = 0.022) and OS (*p* = 0.005; *p* = 0.026) (Fig. 2e–h). However, no correlation was identified between high CD163/CD68+ ratios in both TS and TN with RFS (*p* = 0.085, *p* = 0.782) or OS (*p* = 0.102, *p* = 0.891) (Fig. 2i–l).

## Discussion

TAMs can contribute to tumor destruction and influence tumor growth and progression. M1-like macrophages, characterized by CD68 expression, produce free radicals that can lead to DNA damage with the potential to contribute to tumoricidal activity. In contrast, M2-like macrophages, characterized by both CD68 and CD163 expression, are considered to promote tumor growth and metastasis by releasing chemokines, which are inflammatory growth factors [1–3]. Even so, the prognostic significance of localizations and densities of CD68+ and CD163+ TAMs in TNC is not well understood.

In our study of TNC, no correlation was found between CD68+ TAMs in TS and TN with any clinicopathological findings, OS or RFS by univariate analysis. CD68 is a pan-macrophage marker as it stains both M1-like and M2-like TAMs. Controversy remains over the role of CD68 in cancer. CD68+ TAMs correlated with favorable prognosis in several organs, such as prostate [20], lung [21], and brain tumors [22]. In contrast, poor prognosis was reported in uterine cervix [23], and bladder carcinomas [24]. Furthermore, earlier studies report that a high density of CD68+ TAMs infiltration in invasive breast cancer was associated with higher vascularity and nodal metastasis, as well as reduced RFS and OS [25, 26]. Also, Tsutsui et al. reported that a high density of CD68+ TAMs had significantly worse disease-free survival [18]. Further, Mahmoud et al. reported on CD68+ TAMs using a large cohort of patients. In their univariate analysis, a high density of CD68+ TAMs predicted worse breast cancer specific survival and shorter disease-free interval [27]. These results suggest that CD68+ TAMs induce an immune response that supports tumor invasion. However, similar to our findings, Medrek et al. found that CD68+ TAMs showed no correlations between clinicopathological findings and RFS and OS in TNC [28]. Recently, Yang et al. reported that CD68+ TAMs in TNC were not associated with RFS or OS in multivariate analysis [7]. These results suggest that CD68+ TAMs are not an important prognostic factor for patients; however, these results are probably due to CD68 expressing both M1-like and M2-like TAMs, which have opposing effects.

CD163, a well-known specific marker for M2-like macrophages, was found to be closely correlated with unfavorable prognostic factors in several studies [8–10, 29, 30]. Medrek et al. reported that TNC showed more TAMs infiltration, especially CD163+ cells, than other types of breast cancers [28]. However, they did not find any prognostic significance of CD163+ TAMs in TN. Further, Yang et al. found that increased CD163+ TAMs in TS were correlated with unfavorable clinicopathological factors, and worse RFS and OS [7]. However, they did not find any statistical difference in CD163+ TAMs in TN. Several studies in breast cancer have reported the locations of TAMs [7, 19, 27, 28]. Therefore, we also used full block-face tissue sections to estimate TAMs in TS and TN separately to assess their prognostic value in our TNC cohort. We found in multivariate Cox regression analyses using the median as the cut-off that CD163+ TAMs in both TS and TN were independent prognostic factors for worse RFS and OS. From these results, it is suggested that CD163+ TAMs affect the prognosis of TNC by not only regulating the immune reaction by TAMs in TS, but also through their direct influence on TN.

We also examined the correlation between TAMs and TILs which have recently been highlighted as prognostic markers and potential targets for adjuvant therapy [31–34]. TILs have antitumor activity and a favorable prognostic effect in breast cancer, especially in TNC [16, 35–37]. In our study, no significant correlations were found between the infiltration densities of TAMs and TILs. However, we could not draw any conclusion on the basis of our small number of cases.

There is a limitation in this study. First, the methods and subtypes of breast cancer patients were different in other studies, including our own. Yang et al. examined cases in which Basal-like carcinoma was defined by not only TNC, but also by EGFR and/or CK5/6 expression [7]. Second, although CD163 is regarded as a highly specific M2 macrophage marker, it can also be expressed by myeloid dendritic cells (MDCs). Both macrophages and MDCs are members of the mononuclear phagocyte system, these cells are considered distinct cell types based on their morphology and functions. Macrophages are defined as large vacuolar cells that have oval or rounded nuclei, while MDCs are characterized as stellate migratory cells. Therefore, we could have excluded the majority of the CD68+ and CD163+ MDCs with morphological features. Nevertheless, it could not be confirmed whether or not CD68+ and CD163+ MDCs are located in TS and TN. Of the different cell characteristics, surface markers are often used to distinguish MDCs from macrophages, but phenotypic analysis has been considered insufficient to define MDCs subsets. Some specific markers have been suggested to detect M1/M2 macrophages, but they remain controversial. In the future, more studies on larger sample sizes and TAMs labeling new, reliable macrophage markers are needed to evaluate the clinical value. Further, Medrek et al. observed some CD163+ areas that lacked CD68 expression. They suggested this result was due to a CD163-expressing subset of immature myeloid cells with prognostic impact [28]. Here, we confirmed TAMs not only by immunohistochemical staining, but also H&E staining, then estimated the number of typical macrophages and excluded the possibility that MDCs cells or myeloid-derived cells expressed CD163. However, further investigation is needed to identify TAMs’ roles in TNC with new, specific markers in future studies.

## Conclusions

We examined the prognostic value of TAMs in TNC. Our study found that infiltration of CD163+ TAMs, rather than CD68, in both TS and TN was associated with poor prognosis in TNC patients by multivariate analysis. This suggested that CD163+ TAMs may affect the prognosis of TNC by not only regulating the immune reaction by TAMs in TS, but also through their direct influence on TN.
